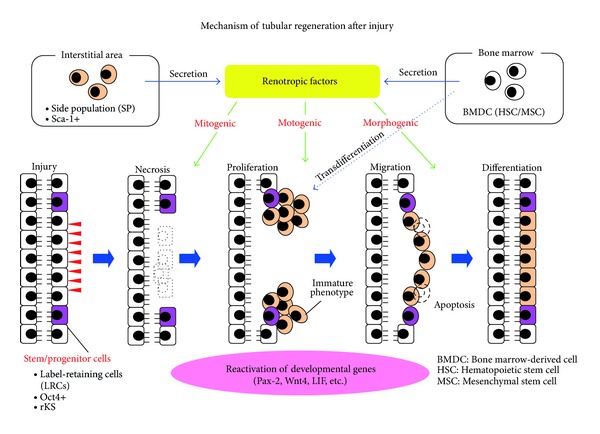# Regenerative Medicine for the Kidney: Renotropic Factors, Renal Stem/Progenitor Cells, and Stem Cell Therapy

**DOI:** 10.1155/2014/595493

**Published:** 2014-05-08

**Authors:** Akito Maeshima, Masao Nakasatomi, Yoshihisa Nojima

**Affiliations:** Department of Medicine and Clinical Science, Gunma University Graduate School of Medicine, 3-39-15 Showa, Maebashi, Gunma 371-8511, Japan

## Abstract

The kidney has the capacity for regeneration and repair after a variety of insults. Over the past few decades, factors that promote repair of the injured kidney have been extensively investigated. By using kidney injury animal models, the role of intrinsic and extrinsic growth factors, transcription factors, and extracellular matrix in this process has been examined. The identification of renal stem cells in the adult kidney as well as in the embryonic kidney is an active area of research. Cell populations expressing putative stem cell markers or possessing stem cell properties have been found in the tubules, interstitium, and glomeruli of the normal kidney. Cell therapies with bone marrow-derived hematopoietic stem cells, mesenchymal stem cells, endothelial progenitor cells, and amniotic fluid-derived stem cells have been highly effective for the treatment of acute or chronic renal failure in animals. Embryonic stem cells and induced pluripotent stem cells are also utilized for the construction of artificial kidneys or renal components. In this review, we highlight the advances in regenerative medicine for the kidney from the perspective of renotropic factors, renal stem/progenitor cells, and stem cell therapies and discuss the issues to be solved to realize regenerative therapy for kidney diseases in humans.

## 1. Introduction

**Figure 1:**
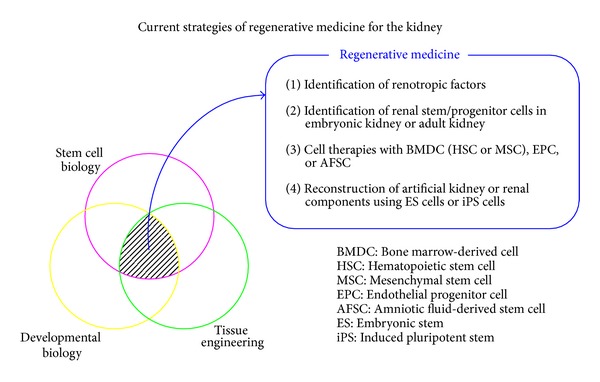


The kidney is indispensable for tissue homeostasis as well as regeneration. Renal tubular epithelium composed of polarized mature cells has the capacity to regenerate following acute kidney injury. After the insult occurs, these cells rapidly lose their brush border and dedifferentiate into a more mesenchymal phenotype. The dedifferentiated cells migrate into the regions where cell necrosis, apoptosis, or detachment has resulted in denudation of the tubular basement membrane. They proliferate and eventually redifferentiate into an epithelial phenotype, completing the repair process [1]. Recent studies suggest that renal stem/progenitor system is present in the tubules, interstitium, and glomeruli of the adult kidney and functions as the main drivers of kidney regenerative responses after injury. Understanding the mechanisms that drive renal progenitor growth and differentiation represents the key step for modulating this potential for therapeutic purposes [2]. However, renal fibrosis, the inevitable consequence of an excessive accumulation of extracellular matrix, is irreversible. Patients with chronic renal disease show a progressive decline in renal function with time, finally leading to end-stage renal failure that requires lifelong dialysis or renal transplantation. Many therapeutic interventions seem to be effective in animal models of acute or chronic kidney injury. Nonetheless, it is still difficult to translate these promising results into humans in the clinical setting. As a new therapeutic option, regenerative therapies for the kidney have been extensively investigated from the aspect of stem cell biology, developmental biology, and tissue engineering. The four major strategies of regenerative medicine for the kidney are as follows: (1) identification of renotropic factors; (2) identification of renal stem/progenitor cells in embryonic kidney or adult kidney; (3) cell therapies with bone marrow-derived cells (BMDCs), namely, hematopoietic stem cells (HSCs) or mesenchymal stem cells (MSCs), endothelial progenitor cells, and amniotic fluid stem cells; and (4) reconstruction of artificial kidney or renal components by using embryonic stem (ES) cells or induced pluripotent stem (iPS) cells (Figure 1). In this review, we highlight the recent advances of regenerative medicine for the kidney from the perspective of renotropic factors, renal stem/progenitor cells, and stem cell therapies and clarify the issues to be solved for the establishment of regenerative therapy.

## 2. Renotropic Factors

The regeneration process resembles the developmental paradigm. The remodeling and maturation of restored epithelium after renal injury have many parallels with the growth and maturation that occur during kidney organogenesis. Soluble factors involved in kidney development have been identified by gene targeting techniques, in vitro tubulogenesis models, and organ culture systems. By using animal kidney injury models, most of these factors also have been proved to regulate kidney recovery as potential renotropic factors. These factors include hepatocyte growth factor (HGF) [3], epidermal growth factor [4], insulin-like growth factor-I (IGF-I) [5, 6], heparin-binding EGF-like growth factor (HB-EGF) [7, 8], platelet-derived growth factor (PDGF) [9], bone morphogenetic protein-7 (BMP-7) [10, 11], and uterine sensitization-associated gene-1 (USAG1), a novel BMP antagonist [12]. Recently, the essential role of their receptors in kidney injury also has been demonstrated. Mice with a specific EGF receptor deletion in renal proximal tubules showed the importance of EGF receptor activation in the recovery phase after acute kidney injury [13]. Conditional knockout mice lacking the HGF receptor,* c-met*, specifically in renal tubules demonstrated the antiapoptotic or anti-inflammatory role of* c-met* signaling in renal protection after acute kidney injury [14]. Deletion of the BMP receptor activin-like kinase 3 (Alk3) in the tubular epithelium enhances TGF-beta signaling, epithelial damage, and fibrosis [15].

A negative regulator of kidney repair has also been identified. Data from transgenic mice expressing truncated activin type II receptor [16], an in vitro tubulogenesis model [17], the Wolffian duct culture [18–21], and isolated rat embryonic kidney culture [20] indicate that activin A is an endogenous inhibitor of renal organogenesis [22, 23]. Additionally, activin A is a potent inhibitor of renal regeneration after injury [24].

Key regulatory molecules required for renal organogenesis are reactivated in regenerating tubular cells after ischemic injury. These factors include Pax-2 [25–27], leukemia inhibitory factor [28], and Wnt4 [29].

Although many renotropic factors or signaling pathways have been identified, the mechanism by which these growth factors mediate recovery from renal injury is not totally understood. Most of these factors are epithelial cell mitogens in vitro, and they induce tubular cell proliferation after injury when exogenously administered. However, it remains unknown if these factors are involved in cell maturation, restoration of polarity, modulation of renal blood flow, and neutrophil infiltration. It is of great interest to examine if these renotropic factors promote renal regeneration via the activation of intrinsic renal stem cells. Recently, a critical role of peritubular capillary endothelium as a source of factors required for tubular recovery after injury has been reported [30]. Mechanisms of cell-cell interactions such as tubular epithelium and peritubular capillary endothelium or interstitial fibroblasts need to be clarified.

## 3. Renal Stem/Progenitor Cells

Despite the structural complexity of the adult kidney, attempts to identify adult kidney stem cells have been made based on the broad principles of stem cell biology, such as prolonged cell-cycling time (label-retaining cells), Hoechst dye extrusion (side population cells), by growth in restrictive cell culture conditions, or expression of markers for other tissue stem cells or embryonic kidney.

### 3.1. Identification of Renal Stem/Progenitor Cells Based on Cell Behavior

Stem cells are considered to have an inexhaustible capacity for self-renewal and differentiation to ensure the lifelong maintenance of tissue homeostasis. To conserve growth potential and prevent genetic injury during mitosis, stem cells cycle slowly and are recruited only as demanded by tissue turnover.

One of the most common methods to identify stem cells is to search for slow-cycling cells by labeling their DNA with 5-bromo-2-deoxyuridine (BrdU). A pulse of BrdU labeling followed by a chase period allows the detection of slow-cycling label-retaining cells (LRCs), which represent the stem cell compartment. LRCs were identified in renal tubules of normal rat kidneys, and regenerating cells during tubular repair were essentially derived from LRCs [31]. Interestingly, tubular LRCs were involved in the epithelial to mesenchymal transition during renal fibrosis [32]. In vitro characterization revealed that LRCs are a multipotent cell population with tubulogenic capacity [33]. The number of these LRCs declines with age, leading to reduced regenerative capacity after injury in the aging kidney [34]. Other groups also found LRCs in tubules [35, 36], papilla [37], or renal capsules [38]. The location, properties, and behavior of LRCs after injury differ or remain controversial. This lack of consistency is probably due to differences in the timing or duration of the pulse and the length of the chase. Every tubular cell shares the capacity to retain BrdU and proliferate after injury. Nonetheless, the patterns of growth and differentiation of LRCs should be clarified in detail, because factors that can activate LRCs may possess renoprotective effects.

The ability of hematopoietic stem cells to efflux dyes such as Hoechst 33342 and Rhodamine 123 has been used as the basis of a single-step HSC isolation protocol [39]. These side population cells with the same efflux profile were found in the adult rodent kidney. Renal side population cells possess multilineage capacity [40, 41], and the introduction of side population cells into a model of acute experimental renal damage was therapeutically beneficial [40, 41]. In contrast, renal side population cells have no capacity to transdifferentiate into renal cells in vivo [42]. These data remain contradictory in terms of the relative size, origin, and lineage capacity of the renal side population cells. The definition of a marker phenotype that allows isolation without the assessment of dye efflux will be needed.

### 3.2. Identification of Renal Stem/Progenitor Cells Based on Specific Marker Expression

A subset of parietal epithelial cells localized to the urinary pole of Bowman's capsule was identified in human adult kidneys based on coexpression of CD24 and CD133, which are both used as markers of adult tissue stem cells. These cells exhibited multidifferentiation potential and long-term proliferative capacity in vitro. Injection of CD24/CD133 double-positive cells into mice with acute renal failure induced a complete recovery of renal function and restoration of tubular structures [43]. CD24/CD133 double-positive cells with stem cell properties were also found in embryonic kidney [44] as well as in proximal tubules [45]. Their proliferation rate and differentiation capacity into renal epithelial cells seem to be regulated by Toll-like receptor 2 [46, 47]. It is unknown whether these cells elicit repair via functional integration or humoral induction when delivered into the recipient animal. Glomerular hyperplastic lesions have been shown to be derived from the proliferation of CD133/CD24 double-positive cells [48].

CD133 is mainly known as a marker of HSC and endothelial progenitors [49], but recent reports indicate its expression in adult tissue stem cells. A rare population of CD133-positive cells was found in the interstitium, glomeruli, and tubules. When injected into mice with glycerol-induced acute renal injury, CD133-positive cells homed to the kidney and integrated into proximal and distal tubules during the repair process [50].

A nontubular multipotent stem/progenitor cell population was isolated from the adult mouse kidney and characterized as Sca-1 positive [51]. These cells were capable of differentiation into myogenic, adipogenic, and neural lineages. When injected directly into the renal parenchyma after ischemic injury, renal Sca-1-positive cells adopt a tubular phenotype and potentially could contribute to kidney repair.

### 3.3. Identification of Renal Stem/Progenitor Cells Based on Selective Culture Conditions

A unique population of cells that show self-renewal for more than 200 population doublings without evidence of senescence was isolated from rat kidneys. These cells express endothelial, hepatocyte, and neural markers, suggesting the plasticity of these cells. When injected intra-arterially after renal ischemia, these cells differentiate into renal tubules [52]. Screening of stem cell potential in nephron segments revealed that a cell line derived from the S3 segment of the proximal tubules could be maintained for a long term without transformation and replaced partly in injured tubules when engrafted to the kidney after renal ischemia [53]. A rare population of cells expressing several stem cell markers was selectively identified in the interstitium of the medulla. Intrarenal injection of this population into mice with ischemic injury repaired renal damage [54].

## 4. Stem Cell Therapy

BMDCs have a surprising degree of plasticity and differentiate into cell types of multiple organs of the body [55, 56]. Bone marrow (BM) transplantation is commonly used to study BM cell plasticity. The host BM is replaced by donor BM, and after BM chimerism is established, donor cells are tracked in the target tissues. The donor BMDCs are distinguished from host cells by virtue of their chromosome content (male Y chromosome-positive cells in a female host), the expression of a reporter molecule (beta-galactosidase, luciferase, and enhanced GFP), or the performance of a function (reestablishment of a function in a knockout mouse model). BMDCs have the ability to move to distant sites within the body. As in most organs, BMDCs appear in the kidney in response to renal injury. BMDCs can transdifferentiate into renal tubular epithelial cells [57–59], mesangial cells [60–63], glomerular endothelial cells [64, 65], and even podocytes [66, 67]. Based on these data, cell therapy with BMDCs (HSCs and MSCs) has been extensively examined and reported to be effective. In light of their ease of accessibility, BMDCs are strong candidates for the cell source in stem cell therapy.

### 4.1. HSCs

HSCs are undifferentiated cells capable of self-renewal and stepwise differentiation into fully specialized cells of the blood such as erythrocytes, thrombocytes, and leukocytes. BMDCs significantly contribute to the regeneration of the renal tubular epithelium, differentiate into renal tubules [57–59], or promote proliferation of both endothelial and epithelial cells after injury [68]. These data suggest that the enhancements of the mobilization, propagation, and delivery of BMDCs to the kidney hold potential as entirely new approaches for the treatment of acute kidney injury. Stem cell factor and granulocyte colony-stimulating factor (G-CSF) induced HSC homing to the injured kidney, leading to the significant enhancement of the functional recovery of the kidney [69, 70]. In contrast, data against the use of granulocytosis-inducing HSC mobilization protocols for the treatment of ischemic injury was also reported. Unlike the reports above, the boosting of peripheral stem cell numbers was associated with increased severity of renal failure and mortality. High numbers of activated granulocytes seem to obscure the potential renoprotective effects of HSC [71]. There are several reports against the potential of BMDCs to transdifferentiate into tubular cells after injury [72]. Based on the data from transgenic mice that express GFP in BMDCs [73], in mature renal tubular epithelial cells [74], or in all mesenchyme-derived renal epithelial cells [75], it was suggested that, while BMDC recruitment occurs, kidney repair is predominantly elicited via proliferation of endogenous renal cells. BMDCs might contribute to the regenerative process by producing protective and regenerative factors, rather than by differentiating to directly replace damaged cells [75].

The contradictory results in the localization of BMDCs and the degree of the BMDC contribution to kidney regeneration after injury may be due to methodological limitations in tracking BMDCs, particularly in injured tissues. There are differences in the protocols used in these studies (species, type of injury, transplantation methods, type of cells used for transplantation, and specificity and sensitivity of the detection methods for BM cell origin). Cell fusion may also explain this discrepancy [76, 77]. In the reports of bone marrow recruitment to damaged kidneys, the lineage of the recruited BMDCs has not been established. Whether the recruitment of BMDCs has a beneficial effect on chronic renal damage remains unsolved.

### 4.2. MSCs

The other possible candidate for the BM cell responsible for ameliorating renal damage is the MSC. MSCs are undifferentiated adult stem cells of mesodermal origin that have the capacity to differentiate into a range of mesenchymal tissue types, including cartilage, bone, muscle, stroma, fat, tendon, and other connective tissues. MSCs represent a very small fraction of BM cells, but they can be isolated and expanded with high efficiency in culture as plastic adherent cells.

The therapeutic effect of MSC delivery has been demonstrated in animal models of renal damage [78] such as acute kidney injury induced by cisplatin [79, 80], gentamicin [81], intramuscular injection of glycerol [82], or ischemia [83], Adriamycin-induced nephrotic syndrome [84], mesangioproliferative anti-Thy1.1 glomerulonephritis [85], a mouse model of Alport disease [86], glomerular injured athymic mice [87], a rat remnant kidney [88], and a rat kidney transplantation model of chronic allograft nephropathy [89]. Beneficial effects of MSC are primarily mediated via paracrine factors [90] such as VEGF, HGF, IGF-I [91–93], and erythropoietin [94]. Heme oxygenase-1 (HO-1) [95], the SDF-1-CXCR4/CXCR7 axis [96], and CD44/hyaluronic acid interactions [97] play an important role in MSC-mediated protection. On the other hand, maldifferentiation of intraglomerular MSC into adipocytes accompanied by glomerular sclerosis was observed [98].

Adipose tissue-derived stem cells are an attractive source of stem cells with regenerative properties that are similar to those of BMDCs. Adipose tissue-derived stem cell therapy minimized kidney damage or improved renal dysfunction after renal damages such as ischemic injury [99], a mouse progressive renal fibrosis model [100], acute kidney injury induced by cisplatin [101] or folic acid [102], atherosclerotic renal artery stenosis in pigs [103, 104] or swine [105], and a rat antiglomerular basement membrane disease [106]. Beneficial effects of kidney-derived MSCs [107], allogeneic fetal membrane-derived MSCs [108], and human embryonic MSCs [109, 110] against renal damage have been reported.

### 4.3. Endothelial Progenitor Cells (EPCs)

EPCs participate in the repair of tissues, including the kidney, under diverse physiological and pathological conditions. Renal ischemia rapidly mobilizes EPCs, and transplantation of EPC-enriched cells from the medullopapillary parenchyma provided partial renoprotection after renal ischemia [111]. Acute but not chronic elevation of uric acid acts as an endogenous mediator of EPC mobilization and renoprotection [112]. In a chronic renal artery stenosis model, a single intrarenal infusion of autologous EPCs preserved microvascular architecture and decreased microvascular remodeling by preserving hemodynamics [113]. Manipulation of homing signals may potentially allow therapeutic opportunities to increase endogenous EPC recruitment [114].

### 4.4. ES Cells

ES cells with unlimited developmental potential have been induced to differentiate in vitro into a broad spectrum of specialized cell types and are regarded as new tools for the elucidation of disease mechanisms. The generation of ES cell-derived progenitors offers the potential for regenerative therapies. Although kidney structures are complex, differentiation of ES cells into renal epithelial cell lineages has been successfully demonstrated [115–118]. Recently, it was reported that decellularization of intact rat kidneys in a manner that preserved the intricate architecture allowed seeded ES cells to populate and proliferate within the glomerular, vascular, and tubular structures [119].

### 4.5. iPS Cells

Forced expression of selected transcription factors can transform somatic cells into ES cell-like cells, termed iPS cells [120], which have the potential for multilineage differentiation and provide a resource for stem cell-based treatment. Recently, unique methods for stimulating the differentiation of human iPS cells into kidney lineages [121–123] or three-dimensional structures of the kidney [124] have been developed.

iPS cells from normal human mesangial cells [125], renal tubular cells present in urine [126, 127], and fibroblasts of patients with autosomal dominant polycystic kidney disease [128] have been established. Reprogrammed kidney iPS cells may aid the study of genetic kidney diseases and lead to the development of novel therapies.

The therapeutic effect of iPS cells on renal ischemia was also reported. Transplantation of iPS cells reduced the expression of oxidative substances, proinflammatory cytokines, and apoptotic factors and eventually improved survival in rats with ischemic acute kidney injury [129].

### 4.6. Human Amniotic Fluid Stem Cells

Human amniotic fluid stem cells, a novel class of broadly multipotent stem cells that exhibit characteristics of both embryonic and adult stem cells, have been regarded as a promising candidate for stem cell therapy [130]. Beneficial therapeutic effects of amniotic fluid stem cells have been shown in kidney injury models including acute kidney injury induced by glycerol [131, 132] or cisplatin [133], a mouse model of Alport syndrome [134], and a mouse unilateral ureteral obstruction (UUO) model [135].

## 5. Conclusion

In this review, the role of renotropic factors and intra- or extrarenal stem cells in kidney regeneration after injury is summarized (Figure 2). Compared to other organs, data regarding renal stem/progenitor cells remain at a preliminary stage. The precise location, size of the pool, and cellular morphology are either unknown or controversial.

The delivery of soluble factors with the potential to improve the ability of the tissue to repair itself is the most pharmacologically attractive strategy for organ repair in situ. In this regard, clarification of the factors or the signaling pathways that enhance the regenerative capacity of stem/progenitor cells will lead to a better understanding of the mechanisms of kidney regeneration, as well as to the identification of novel therapeutic strategies to facilitate renal repair after acute kidney injury in humans. Considering the recapitulation of the developmental process in kidney regeneration, such factors may be produced by the embryonic kidney. Understanding the molecular basis of kidney development will help us to develop regenerative therapies for kidney diseases.

## Figures and Tables

**Figure 2:**